# Impaired Local and Long-Range Brain Connectivity and Visual Response in a Genetic Rat Model of Hyperactivity Revealed by Functional Ultrasound

**DOI:** 10.3389/fnins.2022.865140

**Published:** 2022-03-24

**Authors:** Marine Droguerre, Benjamin Vidal, Marco Valdebenito, Franck Mouthon, Luc Zimmer, Mathieu Charvériat

**Affiliations:** ^1^Theranexus, Lyon, France; ^2^CNRS, UMR 5292, INSERM U1028, Lyon Neuroscience Research Center, Université de Lyon, Université Claude Bernard Lyon 1, Lyon, France; ^3^CERMEP-Imaging Platform, Bron, France; ^4^Hospices Civils de Lyon, Lyon, France

**Keywords:** functional ultrasound imaging, genetic CNS model, hyperactivity, functional connectivity, visual stimulation

## Abstract

Attention-Deficit hyperactivity disorder (ADHD) is a central nervous system (CNS) disorder frequently associated with other psychiatric disorders. Pathophysiology processes at stake in ADHD are still under investigation and interestingly neuroimaging data points to modulated brain connectivity in patients. The genetic spontaneously hypertensive rat (SHR) model has been widely used to study pathophysiological underpinnings of ADHD and resting-state brain connectivity using functional magnetic resonance imaging. Here, functional ultrasound imaging, a new technique enabling fast measurement of cerebral blood volume (CBV), was used to further characterize resting-state functional connectivity – at both local and long-range – and visual response in SHR. We demonstrated that response to visual stimulation was increased in SHR in the visual cortex and the superior colliculus. They displayed altered long-range functional connectivity between spatially distinct regions. SHR also displayed modulated local connectivity, with strong increases of regional homogeneity in parts of the motor and visual cortex, along with decreases in the secondary cingulate cortex, the superior colliculus and the pretectal area. As CBV is intricately coupled to cerebral activity, these results suggest an abnormal neural activity in the SHR animal model, consistent with previous clinical studies and demonstrate the potential of functional ultrasound imaging as a translational tool in ADHD.

## Introduction

Attention-Deficit hyperactivity disorder (ADHD) is a common neurodevelopmental disorder characterized by a persistent pattern of inattention, impulsivity, and/or hyperactivity (Faraone et al., 2015). Based on the 2021 World Federation of ADHD International Consensus Statement, ADHD is caused by the combined effects of many genetic and environmental risks and occurs in 5.9% of youth and 2.5% of adults (Faraone et al., 2021). A growing number of neuroimaging studies on ADHD highlight various functional alterations in patients; however, they are endowed with some conflicting results (Samea et al., 2019; Cortese et al., 2021). Although no animal model could perfectly reflect this pathology, the spontaneously hypertensive rat (SHR) is the most widely studied genetic rat model of ADHD (Okamoto and Aoki, 1963; Russell, 2011) and exhibits several behavioral characteristics of the disease, especially poor performance in tasks linked to attention, impulsivity, and hyperactivity (Bull et al., 2000; Sagvolden, 2000).

In addition to behavioral and genetic parallels to ADHD, neuroimaging studies revealed that SHR exhibit brain alterations similar to ADHD. This animal model not only presents morphological differences such as lower volumes in various brain areas (Sabbatini et al., 2000; Mignini et al., 2004), but also an altered activity and functional connectivity (Poirier et al., 2017; Zubcevic et al., 2017) in comparison to its control strain, the Wistar-Kyoto rat (WKY). Nevertheless, neuroimaging studies of SHR and WKY rats have been largely restricted by the low spatiotemporal resolution and sensitivity of positron emission tomography (PET) and functional magnetic resonance imaging (fMRI) in small animals (Temma et al., 2008; Huang et al., 2016).

The last years have seen the emergence of functional ultrasound imaging (fUS) (Mace et al., 2011; Deffieux et al., 2021), a new neuroimaging modality allowing to locally monitor cerebral blood volume (CBV) dynamics to indirectly measure brain activation through neurovascular coupling. Based on ultrasonic plane wave emissions, fUS imaging enables to visualize CBV changes at a higher spatiotemporal resolution and sensitivity as compared to fMRI and PET (Urban et al., 2017; Deffieux et al., 2018, 2021). Several works have evaluated the application of fUS technique for studying (i) sensory signal processing (Urban et al., 2014; Gesnik et al., 2017; Rabut et al., 2019), (ii) functional connectivity (Osmanski et al., 2014; Ferrier et al., 2020), and (iii) pharmacological challenge (Mace et al., 2011; Vidal et al., 2020a,b) in different species, from small animals to newborn neonates (Imbault et al., 2017; Rau et al., 2018; Dizeux et al., 2019). Its potential for pathophysiological investigations remains to be further elucidated, and this is one of the scopes of the current study.

In the present study, we investigated, for the first time to our knowledge, the resting-state functional connectivity and response to visual stimuli of the SHR strain compared to WKY, using fUS.

## Materials and Methods

### Animals

In total, nine 8-week-old SHR and nine WKY male rats were used in the study (Janvier Labs, France). They were maintained under controlled environmental conditions (12/12 h light-dark cycle, light on at 7 a.m., 22 ± 1°C ambient temperature, 60% relative humidity) with food and water *ad libitum*. Animal surgery and experimentations were carried out in compliance with the ARRIVE guidelines and were conducted in strict accordance with the recommendations and guidelines of the European Union (Directive 2010/63/EU). Experiments followed the policies of the French Ethic Committee for preclinical research. Procedures and protocols were authorized by the French Ministry of Research (authorization reference: APAFIS#19829). All efforts were made to improve animal welfare and minimize animal sufferings. The sample size of the study was chosen based on our experience on the variability of the technique; we estimated that eight animals per group would be enough to detect changes in functional connectivity or visual response, with one extra per group in case of surgery failure. Two animals were discarded from the WKY group because of hematomas that appeared below the skull after the surgery.

### Skull Thinning

After induction of anesthesia (isoflurane at 4% in 1 L/min air), the rats were transferred to a stereotaxic frame (U-frame, WPI) with continuous delivery of isoflurane at 2% in 0.6 L/min of air through a mask during the surgery. For analgesia, buprenorphine (Buprecare, Axience) was subcutaneously injected at 0.05 mg/kg. After shaving and cleaning with betadine, the scalp was incised, and the skin was pulled to visualize its lateral sides from the bregma to the lambda anatomical landmark. The bone was thinned from +3.00 to –7.00 mm (AP) and +5.00 to −5.00 mm (L) from bregma using a drill at low speed (Harvard Apparatus, 75–1887). Saline was frequently added between drilling sessions to avoid overheating until the skull was thin enough to be flexible. The scalp was then sutured, betadine and lidocaine were locally applied, and the rats were allowed to recover from anesthesia 3 days before the imaging session.

### Functional Ultrasound Imaging

Anesthesia induction was performed using isoflurane at 4%, then lowered to 2% during imaging. The body temperature was monitored with a rectal probe and maintained using a heating blanket, and respiratory and heart rates were continuously monitored (TCMT, Minerve, France). After local application of lidocaine, the thinned skull was exposed and covered with ultrasound gel. The rats were scanned with a system dedicated to small animal ultrasound neuroimaging (Iconeus, Paris, France). Doppler vascular images were obtained using the Ultrafast Compound Doppler Imaging technique (Bercoff et al., 2011). Each frame was a Compound Plane Wave frame (Montaldo et al., 2009) resulting from the coherent summation of backscattered echoes obtained after successive tilted plane waves emissions. A stack of hundreds of such compounded frames was acquired with a very high frame rate. Then, the blood volume signal was extracted from the tissue signal by filtering the image stacks with a dedicated spatiotemporal filter using Singular Value Decomposition (Demene et al., 2015). Each transcranial Power Doppler image was obtained from 200 compounded frames acquired at 500 Hz frame rate.

### Visual Stimulation Protocol

**FIGURE 1:**
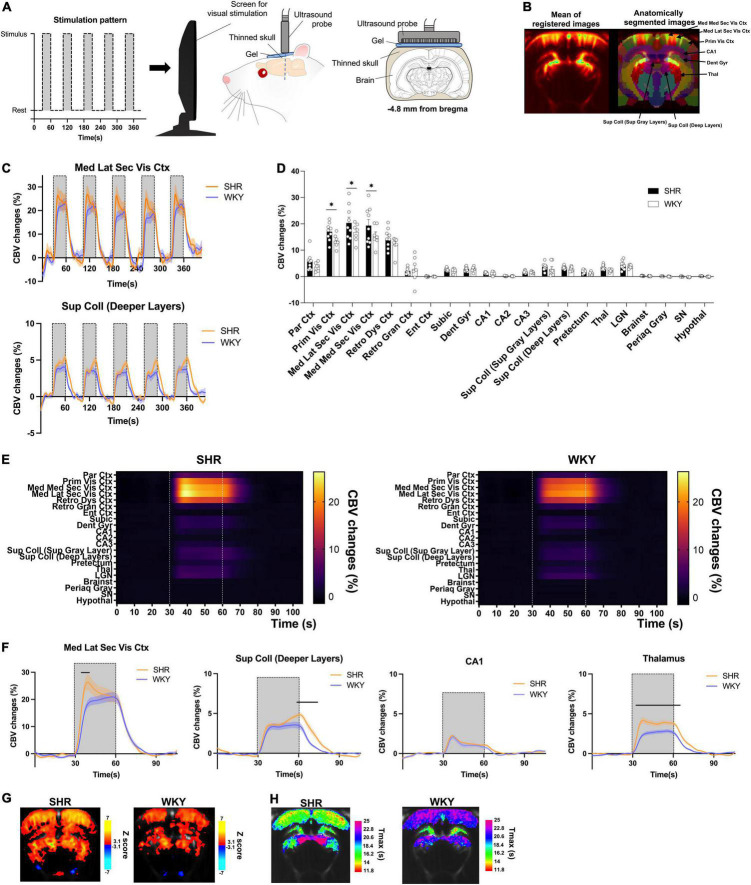
Functional Ultrasound Imaging (fUS) responses to visual stimulation in WKY and SHR rats. Functional ultrasound acquisition during visual stimulation **(A)**. **(B)** Mean of registered images during a fUS session (left) and overlay of an anatomical atlas (right) for all animals. Some regions of interest are shown for better understanding of the main results. **(C)** Time curves of CBV changes in the mediolateral secondary visual cortex (upper) and superior colliculus (lower). Visual stimulation periods are shown by horizontal lines (*n* = 7–9 rats per group; mean ± SEM) **(D)** mean CBV changes during the five stimulation periods in the different regions of interest (mean ± SEM). Two-way ANOVA followed by multiple comparisons correction with an FDR of 0.1 (*). **(E)** Visual response time curves over the five stimulation periods in all ROIs. Visual stimulation periods are shown by vertical dotted lines. **(F)** Mean time curves of CBV changes during all five stimulation periods in the mediolateral secondary visual cortex (top left), superior colliculus (top right), CA1 of the hippocampus (bottom left), and thalamus (bottom right). Significant differences between SHR and WKY rats are show by horizontal bars. Two-way repeated-measures ANOVAs followed by Sidak’s multiple comparisons tests (*p* < 0.05, mean ± SEM) **(G)** statistical pixel-based analysis of the visual stimulation response as compared to non-stimulated period (*p* < 0.001). **(H)** Temporal profile of the visual stimulation response, with a pixel-to-pixel mapping of the mean time to maximal CBV increase during the five stimulations in all animals for each group.

For fUS imaging experiments during visual stimulation, the probe was placed at bregma −4.8 mm (AP) (Figure 1A). The stimuli delivery was adapted from a previous study (Gesnik et al., 2017). Briefly, visual stimuli were delivered using a screen in front of the rat at 8 cm. Stimulation runs consisted of black and white flickering on the screen for stimulation (at random frequencies ranging from 0.5 to 15 Hz) and continuous black screen for rest. The stimulation pattern consisted in 30 s of initial rest followed by runs of 30 s of flicker and 45 s of rest, repeated five times. A Power Doppler image was acquired every 0.4 s during 405 s. This acquisition was performed in the dark after waiting for at least 15 min, to ensure that the rats were dark-adapted. All animals underwent two sessions of visual stimulation at different days, enabling to double the total number of acquisitions to be analyzed (*n* = 9 SHR and *n* = 7 WKY rats were used in this experiment).

### Resting-State Protocol

**FIGURE 2:**
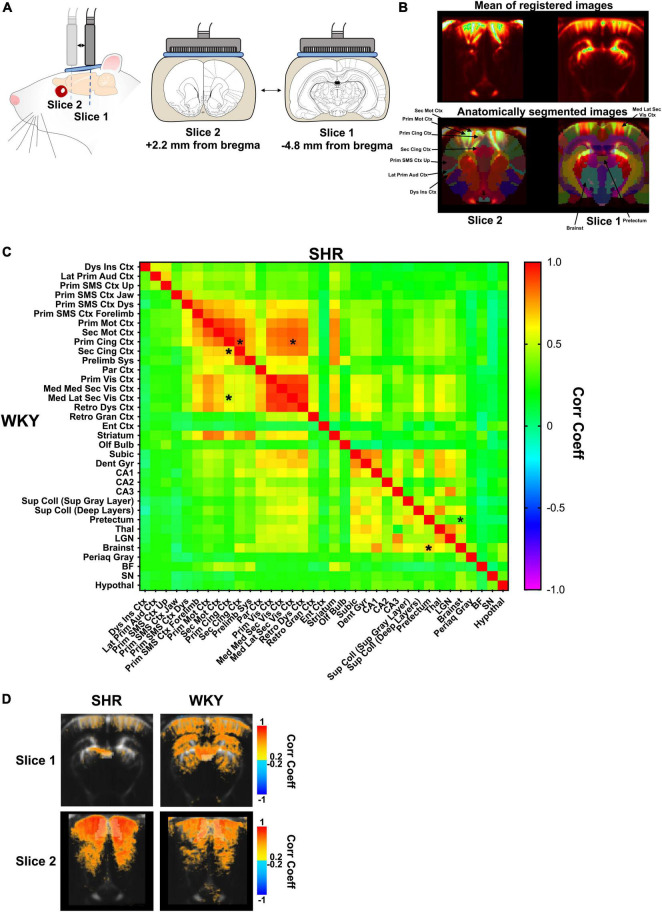
Resting-state long-range functional connectivity in SHR and WKY rats. Functional ultrasound acquisition in the absence of stimulation (i.e., resting-state condition) **(A)**; During resting-state experimentation, the probe alternates between bregma –4.8 mm and bregma +2.2 mm. **(B)** Mean of registered images during a fUS session (upper) and overlay of an anatomical atlas (lower) for all animals. Some regions of interest are shown for a better understanding of the main results. **(C)** Two-way ANOVA followed by multiple comparisons correction with a FDR of 0.1 (*). **(D)** Seed-based functional connectivity maps of slice 1 (top) and slice 2 (bottom) in SHR (left) and WKY (right) rats showing the connectivity with the pretectum (top) or primary cingulate cortex (bottom) with the other pixels on the same slice (average of all animals for each group).

For fUS imaging acquisitions in resting state, the probe was set to continuously move between bregma −4.8 mm and bregma + 2.2 mm (AP) in order to follow CBV changes in the visual areas (slice 1) and in the prefrontal areas (slice 2) (Figure 2A). A Power Doppler image was acquired every 2.8 s in the same plane (with an interval of 1.4 s between the two slices) during 20 min. In total, *n* = 8 SHR and *n* = 7 WKY rats were used in this experiment (one SHR rat was discarded from the analysis because a spontaneous movement was observed during the acquisition, suggesting anesthesia instability). For the slice 2, *n* = 7 for SHR and *n* = 5 for WKY acquisitions were kept, due to transient artifacts that were punctually observed in some acquisitions at this slice (because of the long distance for the probe to make between the two slices).

### Visual Stimulation Data Analysis

Before data processing, all acquisitions were automatically registered together in MATLAB as previously described (Vidal et al., 2021), along with the data from the resting-state protocol that were acquired in the same plane. Briefly, a custom template was obtained by computing the mean image of all acquisitions, generating a first mean reference image, before linear registration of each single initial acquisition on this reference image. A second mean image of all those registered acquisitions was computed to generate a new template before performing linear registration of all initial unregistered acquisitions again. These steps were repeated for several iterations until there was no residual difference between two successive means, meaning that the optimal template was obtained. All fUS acquisitions were registered on this final template using linear registration before non-linear registration using the Demons algorithm (Pennec and Ayache, 1998). This registration method was estimated on singular value decomposition (SVD)-denoised images and applied to the original data. The registered data were then temporally filtered (highpass filter, cut-off frequency of 0.0025 Hz, and Gaussian lowpass filter with a cutoff frequency of 0.0375 Hz at half power point).

The corresponding 2D slice of interest (−4.8 mm from bregma) was extracted from the SIGMA atlas (Figure 1B; Barriere et al., 2019) and coregistered on the fUS template using manual transformations in the ITK-Snap software, until the boundaries of the cortex and bottom of the brain matched those from the template. The Power Doppler signal was then automatically extracted from each region for each individual scan using a MATLAB script and normalized to the rest periods, enabling the measurement of the CBV changes occurring during visual stimulation. The average CBV change during all stimulations was calculated and the five time-courses were averaged to estimate a global CBV time-course during visual stimulation for each acquisition, before averaging all individuals for each group. More details on the visual stimulation data analysis are provided in the Supplementary Material.

### Resting-State Data Analysis

All acquisitions were realigned in the same space as described above. The data acquired in the slice 1 (bregma −4.8 mm) were realigned together with the visual stimulation data as they were obtained in the same coronal plane, and the data acquired in the slice 2 (bregma +2.2 mm) were realigned together as a different set of data.

For the analysis of functional long-range connectivity, the data were temporally filtered (lowpass and highpass filters with respective cutoff frequencies of 0.13 Hz and 0.00067 Hz), before Power Doppler signal extraction from each region for each individual scan using a MATLAB script. ROIs time-courses were reinterpolated and shifted to synchronize the measurements between the two slices. Then, the temporal correlations were measured by the Pearson correlation coefficient for each pair of regions from 1 to 20 min of acquisition to generate a correlation matrix. The first minute of acquisition was discarded from the analysis because of bubbles artifacts that were observed in a few scans (which transiently appeared because of the displacement of the probe through the ultrasound gel). Mean connectivity matrices were estimated for each group. For further illustration, seed-based connectivity maps were generated by measuring the Pearson correlation coefficient between a seed ROI and all the pixels, for each acquisition, before averaging the individuals for each group (Figure 2D).

**FIGURE 3:**
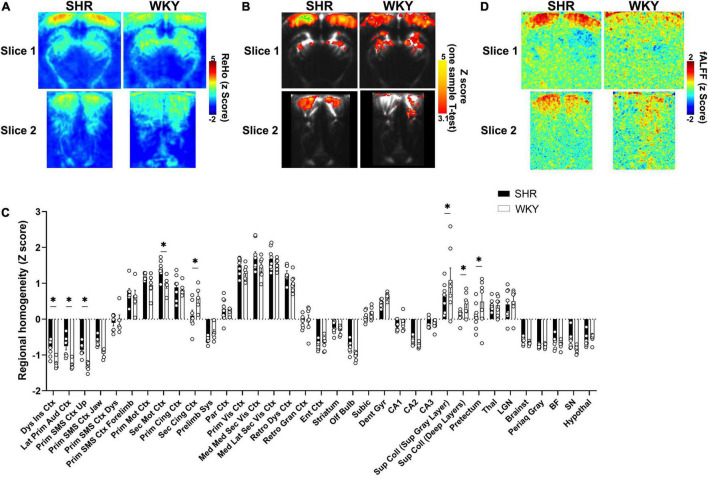
Resting-state local functional connectivity in SHR and WKY rats. Averaged **(A)** and statistical **(B)** maps of resting-state brain regional homogeneity (ReHo) pattern in SHR and WKY rats (*p* < 0.001 in **B**). **(C)** with a FDR of 0.1 (*). **(D)** Averaged map of resting-state fractional amplitude of low-frequency fluctuations (0.01–0.08 Hz).

For the regional homogeneity (ReHo) analysis, the coherence was measured between each pixel and its neighbors, as previously described in detail for fMRI studies (Liu et al., 2010), using a script adapted from the REST toolbox (Song et al., 2011). Briefly, Welch’s method was used to estimate the power spectrum and cross spectrum of any two time series of a pair of pixels, and their coherence was calculated in the 0.01–0.08 Hz band (as usually done in ReHo studies) using their band-averaged estimates of the cross and power spectra. The overall coherence within a given cluster (a pixel and its eight neighbors) was estimated by averaging the coherence obtained across all pairs of pixels in the cluster, and was assigned to the center pixel. For normalization purposes and as previously suggested (Liu et al., 2010), each ReHo map was expressed as Z scores by subtracting each pixel value to the global mean in the brain slice, before division by the standard deviation. Mean ReHo images were generated for each group of animals. Statistical maps of ReHo (Figure 3B) were estimated with SPM12 using one-sample *T*-tests for each group after Gaussian smoothing (0.3 mm × 0.3 mm FWHM). The mean ReHo values in the ROIs were also extracted and compared between groups. More details on the resting-state data analysis are provided in the Supplementary Material.

### Statistical Analysis

For the ROIs analyses, two-way ANOVAs were used to compare mean CBV changes during visual stimulation, coefficient correlation values from the functional connectivity analysis (after Fisher *Z*-transformation), and ReHo values (after conversion to Z-scores), followed by multiple comparisons with an FDR of 0.1. For the comparisons of the ROIs time-courses during stimulation, two-way repeated-measures ANOVAs were used followed by Sidak’s multiple comparisons tests (*p* < 0.05). For the pixel-based analyzes, the T-maps obtained in SPM were converted into Z-maps using MATLAB and the statistical significance was set at *p* < 0.001.

## Results

### Response to Visual Stimulation Is Increased in Spontaneously Hypertensive Rats With Specific Spatiotemporal Patterns

Spontaneously hypertensive rat and Wistar-Kyoto rat underwent functional ultrasound acquisitions during visual stimulation at bregma −4.8 mm (Figure 1A). Inter-individual registration enabled to perform various analyzes on the fUS data using an anatomical atlas (Figure 1C) or at the pixel level. Both SHR and WKY rats displayed pronounced CBV increases in visual areas such as the visual cortex and the superior colliculus during the stimulation periods. Such responses were very consistent across all five stimulations, as shown in Figure 1C, and we did not observe any change in their shape nor their intensity depending on the order of presentation of the different stimulation blocks, in any region.

As compared to WKY rats, the intensity of the response was increased for several regions of the visual system in SHR animals, as exemplified in the mediolateral visual cortex and the deeper layers of the superior colliculus (Figure 1C). Compared to the rest period, this response was significantly higher in SHR rats in the primary, mediolateral secondary, and mediomedial secondary subdivisions of the visual cortex (+3.5, +3.6, and +3.9% and *p* = 0.0025, *p* = 0.0022, and *p* = 0.0009, respectively; all *q*-values = 0.0178; Figure 1D).

Interestingly, the shape of the response to visual stimulation was also markedly different in all areas of the visual cortex. Indeed, we observed a strong initial increase at the beginning of the stimulation that decreased in intensity during the remaining stimulation period in SHR whereas a slow increase was observed over the stimulation period for WKY animals. On the contrary, in the deeper layers of the superior colliculus, CBV reached a peak at the end of each stimulation period in SHR animals, whereas the response was more stable in WKY rats. The temporal features of the response were investigated on the average time-courses over the five stimulations periods (all regions are shown in Figure 1E). This approach enabled to clearly show that SHR rats displayed an initial peak (from +3.6% to +8.9% compared to WKY, depending on the region) during the visual response in various regions of the cortex (Figures 1E,F), with a significant increase compared to WKY rats in the mediolateral (Figure 1F) and mediomedial secondary visual cortex, the primary visual cortex, the dysgranular retrosplenial cortex and the posterocaudal parietal cortex (around 3–10 s after the beginning of the simulation period).

Some subcortical regions also displayed a significant increase in the initial response, such as the CA2 and CA3 hippocampal subfields, the lateral geniculate nuclei, and the brainstem (Supplementary Figure 1). Interestingly, the opposite was observed in the deep layers of the superior colliculus (Figure 1F), with a similar initial response but a peak occurring at the end of the stimulation period (from the end to around 15 s later) only found in SHR animals, resulting in a significant increase compared to WKY (a maximal increase of +1.9%). In other regions, the visual response was significantly higher in SHR during the whole stimulation period, such as the thalamus (Figure 1F), the pretectum, and even the hypothalamus (maximal increases of +1.9, +0.9, and +0.2%, respectively; (Supplementary Figure 1). Conversely, the mean response was almost identical between the strains in some regions such as the CA1 (Figure 1F), the dentate gyrus and the subiculum (Supplementary Figure 1). Finally, we observed a very slight CBV decrease in the substantia nigra only in WKY rats (maximal decrease of −0.3%).

A pixel-based analysis (Figure 1G) confirmed that SHR rats produced widespread significant CBV responses during the visual stimulations as compared to rest, observed mainly in the cortex and various areas of the midbrain, but also in the hippocampus and the hypothalamus. For WKY rats, the responses were also found in the cortex, midbrain (although mainly in the colliculi) and hippocampus, but clusters were less widespread and less significant. Small negative responses were also found for both strains, located in the ventral regions.

Finally, the temporal profile of the responses was analyzed by mapping pixel-by-pixel the time to maximal CBV increase (here referred to as the Tmax) during the stimulations (Figure 1H). This enabled us to confirm the strong differences between the strains: SHR rats exhibited clear regional differences, with maximal changes that were reached sooner in the cortical areas, especially the most extern layers (Tmax ranging approximately from 11 to 16 s). The lateral geniculate nucleus and the CA1 and dentate gyrus reached maximal changes shortly after, whereas the pretectum and colliculi displayed a clearly different profile, with Tmax higher in these pixels compared to the rest of the slice, consistently with the late peak in these regions (Figure 1F). On the contrary, WKY rats displayed fewer regional variability, with similar Tmax values across the cortical and midbrain areas that were higher than those of SHR rats, consistently with the time-courses of the respective regions (Figure 1F). Only the hippocampal subfields appeared to produce CBV responses with an earlier peak that was very similar to SHR rats, which was also in line with the time-courses (Figure 1F).

### Long-Range Functional Connectivity Between a Subset of Regions Is Disrupted in Spontaneously Hypertensive Rats

Spontaneously hypertensive rat and Wistar-Kyoto rat underwent functional ultrasound acquisitions in the absence of stimulation (in resting-state), with the probe alternating between bregma −4.8 mm and bregma +2.2 mm (Figures 2A,B). Functional connectivity in SHR and WKY rats was assessed over two coronal slices during resting-state in several ROIs from the anatomical atlas (Figure 2C), with some differences between the strains. The connectivity between cortical areas was generally increased in SHR compared to WKY rats, but significant increases were restricted to the connectivity between the primary cingulate cortex and the secondary cingulate cortex (*p* = 0.0002, *q* = 0.0933) or the mediolateral secondary visual cortex (*p* = 0.0003, *q* = 0.0933). Conversely, in the midbrain, the connectivity between the pretectum and the brainstem was significantly decreased in SHR compared to WKY rats (*p* = 0.0005, *q* = 0.0933). The pretectum also tended to display lower connectivity with other regions in SHR rats (Figure 2C).

These differences were also present in the averaged maps of seed-based functional connectivity (Figure 2D). Indeed, the connectivity was globally low between the pretectum and the rest of the brain in SHR rats, whereas it appeared to be in moderate correlation with the cortex, dorsal hippocampus, dorsal thalamus, and colliculi in WKY rats (Figure 2D, top). On the contrary, the mean correlation values between the primary cingulate cortex and the rest of the cortex appeared to be globally higher in SHR rats (Figure 2D, bottom).

### Local Functional Connectivity Is Modified in Spontaneously Hypertensive Rats

A regional homogeneity analysis was conducted on the resting-state data to map the connectivity between each pixel and its immediate neighbors (Figures 3A–C).

Both averaged (Figure 3A) and statistical (Figure 3B) ReHo maps showed a higher functional homogeneity in the cortex of SHR rats, especially the visual cortex, whereas WKY rats tended to display a slightly higher homogeneity in the midbrain. The ROIs analysis (Figure 3C) showed a significant increase of ReHo in various cortical areas of SHR rats, such as the dysgranular insular cortex (*p* = 0.0055, *q* = 0.0275) and lateral primary auditory cortex (*p* = 0.0008, *q* = 0.0181), upper area of the primary somatosensory cortex (*p* = 0.0021, *q* = 0.0181), and the secondary motor cortex (*p* = 0.0055, *q* = 0.0275). An increasing trend was also found in the mediomedial secondary visual cortex (*p* = 0.0262, *q* = 0.1012). Interestingly, the opposite was found for the secondary cingulate cortex (*p* = 0.0016, *q* = 0.0181). There were also significant ReHo decreases in SHR compared to WKY rats in the superior (*p* = 0.0011, *q* = 0.0181) and deeper layers of the superior colliculus (*p* = 0.0143, *q* = 0.0627) and the pretectum (*p* = 0.0038, *q* = 0.0266).

When computing the contributions of the 0.01–0.08 Hz range (Figure 3D), we also found high values of fractional amplitude that were uniformly distributed in the cortex (especially the visual cortex) in SHR rats, whereas the fractional amplitude maps of WKY rats were heterogeneous and the cortex did not consistently display a high fractional amplitude.

## Discussion

Neuroimaging is a widely used translational approach to model central nervous system disorders, better understand pathophysiological processes and evaluate the impact of therapeutics. The scope of this work is to demonstrate the interest of fUS for disease modeling and identification of brain networks in CNS indications, with a focus on SHR rat model.

Based on the principle of the emission of ultrasonic waves, fUS imaging allows the capture of thousands of frames per second to obtain Power Doppler images, directly proportional to the CBV, and indirectly related to the cerebral activity changes (see Deffieux et al., 2018 for a review). It presents higher spatial and temporal resolution as compared to fMRI and PET [∼100 μm and ∼400 ms for fUS; ∼300–400 μm and ∼2 s for fMRI; 1–2 mm and several minutes for PET (Logothetis, 2008; Lancelot and Zimmer, 2010; Markicevic et al., 2021)]. The measurement of CBV changes is also a more straightforward parameter relative to neuronal activity as compared to the fMRI BOLD, enabling a higher sensitivity and specificity, as suggested by recent data (Nunez-Elizalde et al., 2021). fUS imaging has been rarely exploited for characterizing animal models of CNS pathologies. Here we aimed at further understanding the functional changes occurring in SHR rats using fUS imaging and validate the potential of the technique for studying animal models.

SHR model can be associated with various central nervous system (CNS) indications such as KCNQ2-related epileptic encephalopathy (Berg, 2016) or various orphan forms of epilepsy (Russo et al., 2017; Berg, 2018; Becari et al., 2020), however, it is extensively used as the gold standard rodent model of ADHD. This animal model exhibits the behavioral characteristics of the disorder (Sagvolden, 2000) and displays several variations in the *Dat1* gene encoding the dopamine transporter (Mill et al., 2005), known to be related to ADHD (Cook et al., 1995; Faraone et al., 2005; Madras et al., 2005). Finally, conventional neuroimaging studies have shown structural and functional alterations in SHR rats similar to those observed in ADHD (Danker and Duong, 2007; Huang et al., 2016; Ha et al., 2020).

We investigated both the response to visual stimuli and the resting-state functional connectivity of the SHR rat as compared to the control strain WKY using fUS. We demonstrated that (i) the response to visual stimulation is increased in SHR rats with specific spatiotemporal patterns and that (ii) both the long-range and local functional connectivity are disrupted in SHR rats.

As expected, visual stimuli resulted in an increase in the response of visual areas, in particular the visual cortex and the superior colliculus, in both SHR and WKY rats. However, the intensity of the response was higher in SHR animals in those regions. Growing body of evidence suggests that the superior colliculus, a sensory structure related to distractibility and orienting the head and eyes toward stimuli, is dysfunctional in ADHD (see Overton, 2008 for review). In addition, few studies have shown an increase in the response of the visual system in SHR rats or underlines an exaggerated visual responsiveness of the superior colliculus in ADHD rat models (Clements et al., 2014; Brace et al., 2015), in line with our results.

We also found a marked difference between the two strains with respect to the temporal pattern of response to visual stimulation. Depending on the brain region, SHR rats displayed an initial or a delayed peak during the visual response. To our knowledge, there are no studies evaluating the spatiotemporal pattern of a response to visual stimulation in SHR rats. However, our results, showing a slower change in CBV in the visual cortex in the control strain WKY, are in agreement with a previous fUS study showing that peak CBV response happens earlier in the superior colliculus than in the visual cortex (Gesnik et al., 2017).

A meta-analysis of 55 fMRI studies concluded an ADHD-related hyperactivation in the visual, dorsal attention, and somatomotor networks and a hypoactivation in frontoparietal and attentional networks (Cortese et al., 2012). This sensory hyperactivation is proposed in several studies as a compensatory mechanism following hypoactivation of the prefrontal and cingulate cortex in ADHD (see Fassbender and Schweitzer, 2006 for review). Our results from the visual stimulation experiments are therefore consistent with these clinical findings. Interestingly, the local connectivity in cortical regions involved in sensory or motor processing was globally increased in SHR rats, as shown by the ReHo approach (although it did not reach significance in the visual cortex, the fALFF map clearly shows that this region fluctuates in a very homogeneous way in SHR rats compared to WKY). Conversely, the only cortical regions where ReHo was decreased in SHR matched with the regions that are reported to be hypoactivated in ADHD (Fassbender and Schweitzer, 2006), such as the secondary cingulate cortex, the prelimbic system (which corresponds to the prefrontal cortex) and the retrosplenial granular cortex. Deep regions such as the colliculus and pretectum also displayed a decreased ReHo in SHR rats, which suggest that during resting-state, the colliculus and the visual cortex behave differently despite being both hyperactivated during visual stimuli. Functional connectivity analysis between distinct regions also pointed out abnormalities in similar regions, with significant connectivity decrease between the pretectum and the brainstem, and global increase of connectivity across cortical regions, with significant increases between different parts of the cingulate cortex and between the primary cingulate and the mediolateral visual cortices. These results are also coherent with other works using fMRI in ADHD patients (Fair et al., 2012; Saad et al., 2020) and in SHR (Poirier et al., 2017) regarding the disruption of connectivity in the visual system, default-mode network, or other cortical areas. Our findings also emphasize the involvement of other regions that were previously not described in this model, such as the pretectal area, which may be of critical importance since it was significantly impacted during visual stimulation and resting-state (both in terms of local and long-range connectivity).

Our results should be considered in light of some limitations. Only males were used in our experiment in order to limit possible increased variability due to sex differences in the groups. However, future fUS studies should investigate the differences between SHR and WKY in females, as several studies suggested sex differences in the behavior of SHR rats (Berger and Sagvolden, 1998; Dervola et al., 2012). It is important to note that hypertension is generally a confounding factor in the SHR model of ADHD, and we did not monitor the blood pressure during our experiments. However, this symptom only develops in rats 2–4 weeks older than those used in the current study (de Jong et al., 1995), limiting the potential impact of this parameter. Moreover, our experimental conditions (either resting-state or visual stimulation) should not produce significant fluctuations of the blood pressure, minimizing the influence of this parameter on the dynamic processes that we explored. It is also unlikely that basal differences in absolute blood pressure would result in the subtle differences that we found, as both functional connectivity and visual response changes were restricted to certain regions only. Furthermore, our results are in agreement with electrophysiological findings, for instance regarding the superior colliculus visual response (Brace et al., 2015). This study could be replicated in other ADHD models, such as Wistar-Kyoto hyperactive rat (WKHA) rat (Hendley, 2000). Besides, we chose to include all the data to perform our functional connectivity analysis; however, it has recently been suggested in a multicentric fMRI study in mice to evaluate the specificity of functional connectivity by measuring the connectivity between pairs of regions for which a specific connectivity is expected or not, based on anatomical and functional evidence (Grandjean et al., 2020). Using a similar strategy to select only the most biological plausible data from the fUS dataset could improve functional connectivity findings, although the selection criterions should be carefully chosen as functional network differences can be expected in disease models. Finally, animals were anesthetized and the possibility to explore CBV changes in vigil animals could be the next step in the characterization of this model using fUS; nevertheless, the current study still provides strong data regarding strain comparison and potential brain regions involved in ADHD. Importantly, we could establish the spatiotemporal responses occurring during visual stimulation with high precision and low inter-individual variability (as shown in Figure 1F and Supplementary Figure 1), which will contribute to further understanding the functional role of the different brain regions in the pathophysiology of SHR. Indeed, we observed that the shape of time-courses varied greatly with the strain and depending on the region (instead of a global increase in the intensity of the response, we found for instance specific initial peaks in the cortex or late peaks in the superior colliculi in SHR animals, whereas other areas displayed virtually identical time-courses for both strains). This suggests that different visual subnetworks are dysregulated in specific ways in SHR, instead of a global alteration of the whole visual system, that may underpin different behavioral symptoms and possibly involve distinct cellular or molecular mechanisms.

This illustrates the potential of fUS imaging to study subtle changes occurring after a stimulation with a higher temporal resolution and sensitivity as compared to fMRI, while enabling to study multiple areas even in the deepest regions [as opposed to electrophysiology which usually provides limited brain coverage, for instance, the superior colliculus only in Brace et al. (2015)].

In summary, we employed fUS to study the SHR rat, model of ADHD, in comparison to its control strain. We confirmed that SHR rats displayed markedly higher regional CBV in response to visual stimuli and both altered long-range functional and local connectivity. In particular, we reported a strong involvement of visual areas and superior colliculus in SHR rats after visual stimulation, with unique temporal signatures. We also showed a different functional connectivity pattern between the two strains during resting-state, involving mainly visual (and attentional) pathway, and default-mode network. Therefore, our study demonstrates the potential of fUS imaging for investigating animal models of CNS disorders and opening large possibilities for future studies.

## Data Availability Statement

The datasets presented in this article are not readily available because all data are available through Theranexus archive and can be accessed upon request (a signed data-sharing agreement will be required). Requests to access the datasets should be directed to BV, benjamin.vidal@theranexus.com.

## Ethics Statement

The animal study was reviewed and approved by the CELYNE – CEEA 042 and French Ministry of Research.

## Author Contributions

BV and MV performed the fUS experiments. MD and BV analyzed the data and wrote the first draft of the manuscript. MD and MC designed the experiments and managed the project. MD, BV, LZ, FM, and MC reviewed the article. All authors approved the final manuscript.

## Conflict of Interest

MD, BV, FM, and MC are full-time employees of Theranexus company. The remaining authors declare that the research was conducted in the absence of any commercial or financial relationships that could be construed as a potential conflict of interest.

## Publisher’s Note

All claims expressed in this article are solely those of the authors and do not necessarily represent those of their affiliated organizations, or those of the publisher, the editors and the reviewers. Any product that may be evaluated in this article, or claim that may be made by its manufacturer, is not guaranteed or endorsed by the publisher.
