# Supplementary material for: Impaired Local and Long-Range Brain Connectivity and Visual Response in a Genetic Rat Model of Hyperactivity Revealed by Functional Ultrasound
Source: Front Neurosci. 2022 Mar 24;16:865140. doi: 10.3389/fnins.2022.865140 (PMC8987929; doi:10.3389/fnins.2022.865140)
Supplement: Supplementary file 1 [file Data_Sheet_1.docx]

Supplementary Material

Impaired local and long-range brain connectivity and visual response in a genetic rat model of hyperactivity revealed by functional ultrasound

# Supplementary Methods

## Visual stimulation data analysis

Significant CBV changes during visual stimulation were also mapped at the pixel level using the software Statistical Parametric Mapping (SPM12). An isotropic Gaussian filter was applied on the images (0.3 × 0.3 mm^2^ Full Width at Half Maximum - FWHM). A first-level analysis was performed on each scan using a flexible factorial dividing the scan into time-bins of rest or stimulation. The first-level parametric contrast images (comparing the stimulation and the rest time-bins) were then used in the second-level flexible factorial analysis, enabling to quantify at the group level the significant CBV changes.

To compare the temporal profile of the responses, the time to maximal CBV (Tmax) was calculated in each pixel for each stimulation before averaging the values obtained in the five stimulations, in order to generate a global Tmax map for each acquisition. These individual maps were averaged to generate mean Tmax maps for each group.

## Resting-state data analysis

The fractional amplitude of low-frequency fluctuations (fALFF) between 0.01 and 0.08 Hz was also estimated using a script adapted from the REST toolbox (Song et al., 2011), as an additional method of resting-state analysis that has been used for fMRI studies (Zou et al., 2008). After removing the linear trend, the power spectrum was estimated for each pixel and the amplitude was calculated at each frequency. Then, the sum of amplitude across 0.01-0.08 Hz was divided by that across the entire frequency range (0 - 0.178 Hz). Each individual map was expressed as Z scores, and the mean fALFF maps were generated for each group.

# Supplementary Figures
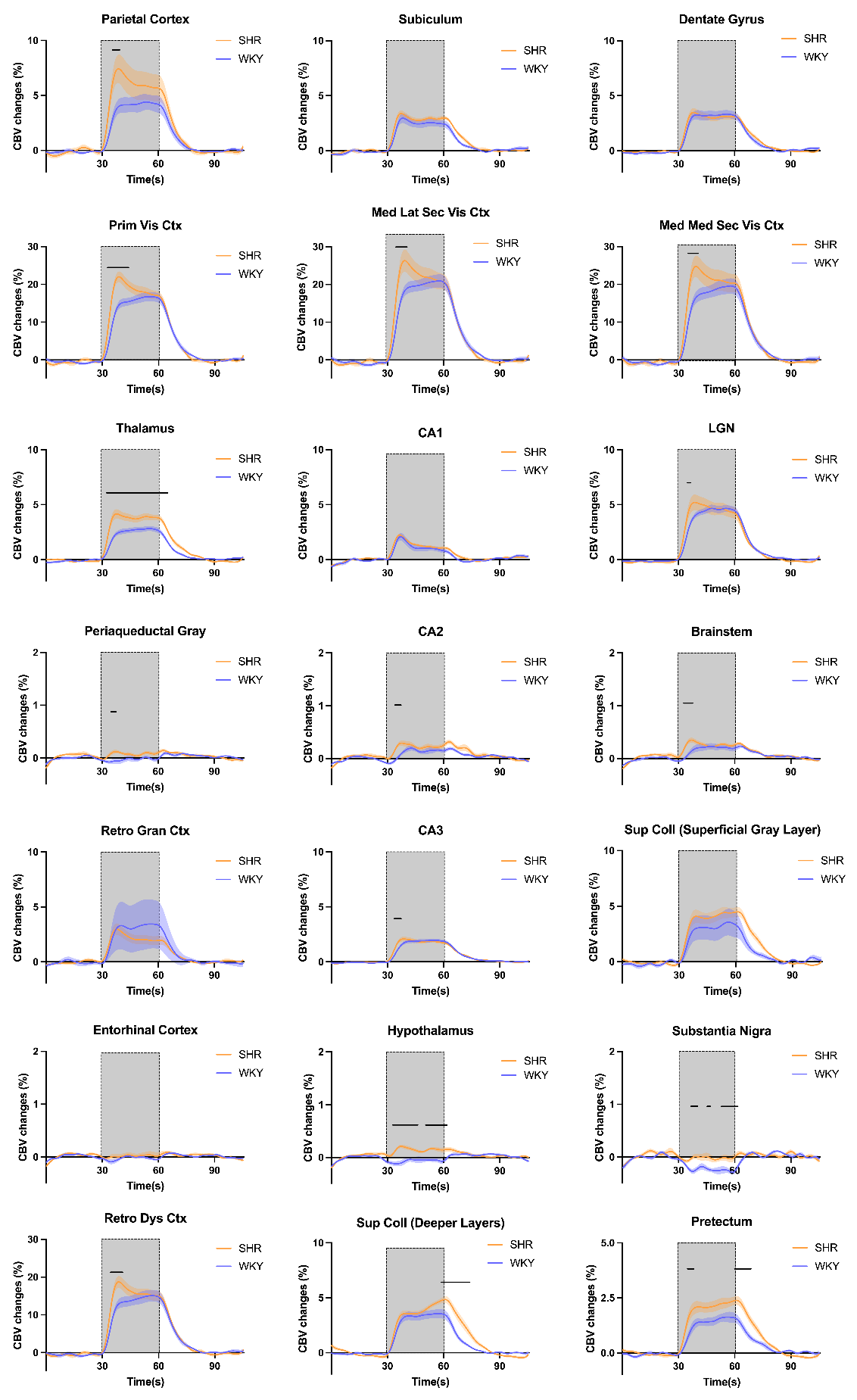


**Supplementary Figure 1.** **Mean time curves of CBV changes during all 5 stimulation periods**. Significant differences between SHR and WKY rats are show by horizontal bars. Two-way repeated measures ANOVAs followed by Sidak’s multiple comparisons tests (p<0.05, mean ± S.E.M). Prim Vis Ctx : Primary Visual Cortex; Med Lat Sec Vis Ctx : Medio Lateral Secondary Visual Cortex; Med Med Sec Vis Ctx : Medio Medial Secondary Visual Cortex; CA1 – CA3 : Cornu Ammonis areas; LGN: Lateral Geniculate Nucleus; Retro Gran Ctx: Retrospenial Granular Cortex; Sup Coll: Superior colliculus; Retro Dys Ctx: Retrosplenial Dysgranular Cortex.

# References

Song, X.W., Dong, Z.Y., Long, X.Y., Li, S.F., Zuo, X.N., Zhu, C.Z., He, Y., Yan, C.G., and Zang, Y.F. (2011). REST: a toolkit for resting-state functional magnetic resonance imaging data processing. *PLoS One* 6**,** e25031.

Zou, Q.H., Zhu, C.Z., Yang, Y., Zuo, X.N., Long, X.Y., Cao, Q.J., Wang, Y.F., and Zang, Y.F. (2008). An improved approach to detection of amplitude of low-frequency fluctuation (ALFF) for resting-state fMRI: fractional ALFF. *J Neurosci Methods* 172**,** 137-141.
